# Histomorphological Spectrum of Cervical Lesions in a Rural Hospital

**DOI:** 10.7759/cureus.18293

**Published:** 2021-09-26

**Authors:** Saravanakumari Vijayakumar, Pammy Sinha, Durga Krishnamurthy

**Affiliations:** 1 Department of Pathology, Sri Lakshminarayana Institute of Medical Sciences, Pondicherry, IND; 2 Department of Obstetrics and Gynaecology, Sri Lakshminarayana Institute of Medical Sciences, Pondicherry, IND

**Keywords:** cervical lesions, rural population, cancer, benign histopathological lesions, chronic cervicitis

## Abstract

Background

Pathologically the cervix is affected by infective, inflammatory, and neoplastic diseases. Non‑neoplastic lesions of the cervix are seen often in sexually active women. Inflammatory lesions include chronic granulomatous cervicitis, acute and chronic cervicitis. In India, cervical cancer is a significant health problem. Many factors contribute to the differences in the spectrum of cervical diseases in the rural population compared to urban areas, but the studies in these populations are scarce.

Materials and methods

A retrospective analysis of all gynecological lesions over one year was studied. All case files were manually extracted, and the data was entered in an Excel sheet. The information included was clinical history (symptoms, signs, menstrual history, duration of illness, parity status), physical examination, per vaginal examination, investigations, including pathological diagnosis. The curated data was then analyzed using IBM SPSS for Windows version 22 (IBM Corp., Armonk, NY).

Results

There were 164 women in the study, with a mean age of 46.07 ± 8.17 years. A majority (n = 124, 75.6%) presented with excessive bleeding. Two-thirds of the study population had a normal cervix on examination. Twenty-seven women had squamous metaplasia, six had low-grade (LSIL) and high-grade squamous intraepithelial lesions (HSIL), and one had malignancy. Excessive bleeding was significantly associated only with LSIL. Among the microscopic findings, only squamous metaplasia (p < 0.001) and dysplasia (p < 0.001) were significantly associated with the final diagnoses, such as LSIL, HSIL, and chronic cervicitis.

Conclusion

Most studies involving rural populations have involved the knowledge, attitude, and practices of the study cohort rather than the histomorphological spectrum of cervical lesions. Since these disorders are also influenced by education, parity, hygiene, and socioeconomic status, it behooves us to be aware of the spectrum of cervical lesions in a rural cohort who differ in these aspects when compared to urban populations. Most of such lesions of the cervix in the population that our medical institution served were benign in nature.

## Introduction

Pathologically the cervix is affected by infection, inflammation, and neoplastic diseases [1]. Non‑neoplastic lesions of the cervix are seen in women of all age groups but are more common in sexually active women [2]. Such non-neoplastic lesions are either inflammatory or tumor-like lesions (Nabothian cysts, endometriosis, endocervical polyp, and endocervical hyperplasia) [2]. Inflammatory lesions are chronic granulomatous cervicitis, acute and chronic cervicitis which may again be infective or non-infective [2]. Carcinoma cervix is usually seen in the 5th decade of life [3]. Worldwide, particularly in developing countries like India, cervical cancer is a significant health problem [3, 4]. Every year, in India, about 90,000 new cervical cancer cases are being reported [3]. In India, carcinoma cervix is diagnosed late, leading to low survival rates. This is due to a lack of screening methods, misconceptions about gynecological diseases, and poor awareness about cervical cancer [4]. The other factors that affect the diagnosis of cervical diseases are age, marital status, education, income, number of children, contraception use, lifestyle, attitude, limited knowledge about cervical carcinoma screening and prevention, less family support, lack of friendly patient-health services [5]. Such factors as listed above contribute to the differences in the spectrum of cervical diseases in the rural population compared to urban areas, wherein the studies involving these populations are scarce.

## Materials and methods

We aimed to describe the histomorphological spectrum of cervical lesions in a rural population of Pondicherry. A retrospective analysis of all gynecological lesions treated in Sri Lakshminarayana Institute of Medical Sciences between April 1, 2020 and March 31, 2021 was performed. The case files and the slides were obtained using the biopsy numbers from requisition forms and then reviewed in the Department of Pathology. After obtaining approval from the Institutional Research Board (No IEC/C-P/13/2021), a search was performed in the Medical Records Department for “Disorders of the cervix” treated in the Department of Obstetrics and Gynecology. All case files were manually extracted, and the data was entered in an Excel sheet. The information included was clinical history (symptoms, signs, menstrual history, duration of illness, parity status), physical examination, per vaginal examination, investigations, including pathological diagnosis. Pap tests data could not be obtained from all patients. The curated data was then analyzed using IBM SPSS for Windows version 22 (IBM Corp., Armonk, NY). Frequencies were calculated for categorical variables and mean ± SD was calculated for continuous variables. Statistical significance was considered if the p-value was ≤0.05.

## Results

There were 164 women in the study, with a mean age of 46.07 ± 8.17 years (Table 1).

**Table 1:** Demographics, clinical, and pathological features Excessive bleeding-one or more of the following: changes of pads more than hourly, the passage of clots, or requirement of blood transfusion

Variable		Frequency (percentage) (n = 164)
Parity	1	6 (3.7)
	2	70 (42.7)
	3	65 (39.6)
	4	15 (9.1)
	5	6 (3.7)
	6	2 (1.2)
Age groups	Young (<41)	46 (28)
	Middle-age (41-60)	111 (67.7)
	Elderly (>60)	7 (4.3)
Symptoms	Uterovaginal prolapse	28 (17.1)
	Leukorrhea	7 (4.3)
	Excessive bleeding	124 (75.6)
Examination	Cervix descended	20 (12.2)
	Keratinized cervix	19 (11.6)
	Recto/cystocele	20 (12.2)
	Decubitus ulcer	9 (5.5)
	Healthy cervix	102 (62.2)
Procedure	Cervix biopsy	56 (34.1)
	Total abdominal hysterectomy	83 (50.6)
	Vaginal hysterectomy	25 (15.2)
Pathological findings	Hyperkeratosis	19 (11.6)
	Epidermidization	13 (7.9)
	Basal cell hyperplasia	6 (3.7)
	Ulcer	9 (5.5)
	Endopapillary cervicitis	18 (11)
	Koilocytosis	15 (9.1)
	Squamous metaplasia	27 (16.5)
	Dysplasia	6 (3.6)
Diagnosis	Nabothian cyst	25 (15.2)
	Polyp	2 (1.2)
	Low-grade squamous intraepithelial lesion	4 (2.4)
	High-grade squamous intraepithelial lesion	2 (1.2)
	Carcinoma cervix	1 (0.6)
	Chronic cervicitis	147 (89.6)

**Figure 1:**
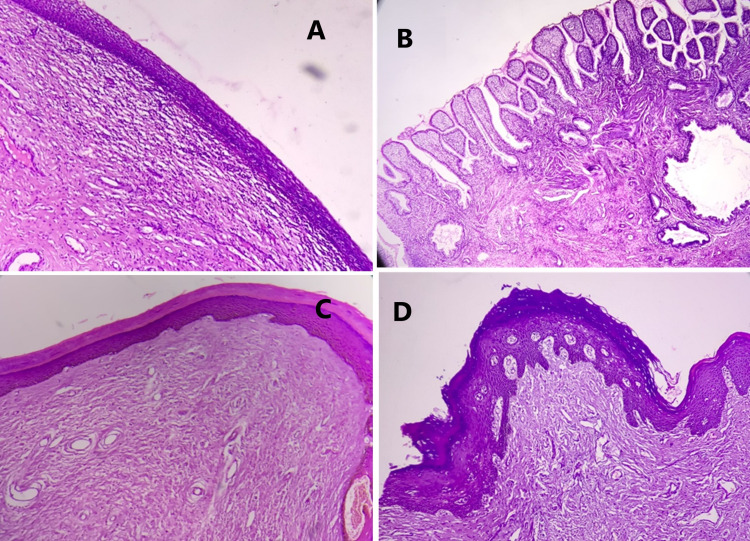
Spectrum of benign histopathological lesions A- H&E stain, 4X: Ectocervix lined by stratified squamous epithelium with sub epithelium showing chronic lymphomononuclear infiltrate suggestive of chronic cervicitis. B- H&E stain, 4X: Cervix shows chronic endopapillary cervicitis with papillary architecture lined by simple columnar epithelium. C and D- H&E stain, 4X: Ectocervix lined by keratinized stratified squamous epithelium shows hyperkeratosis of cervix and epidermidization respectively.

**Figure 2:**
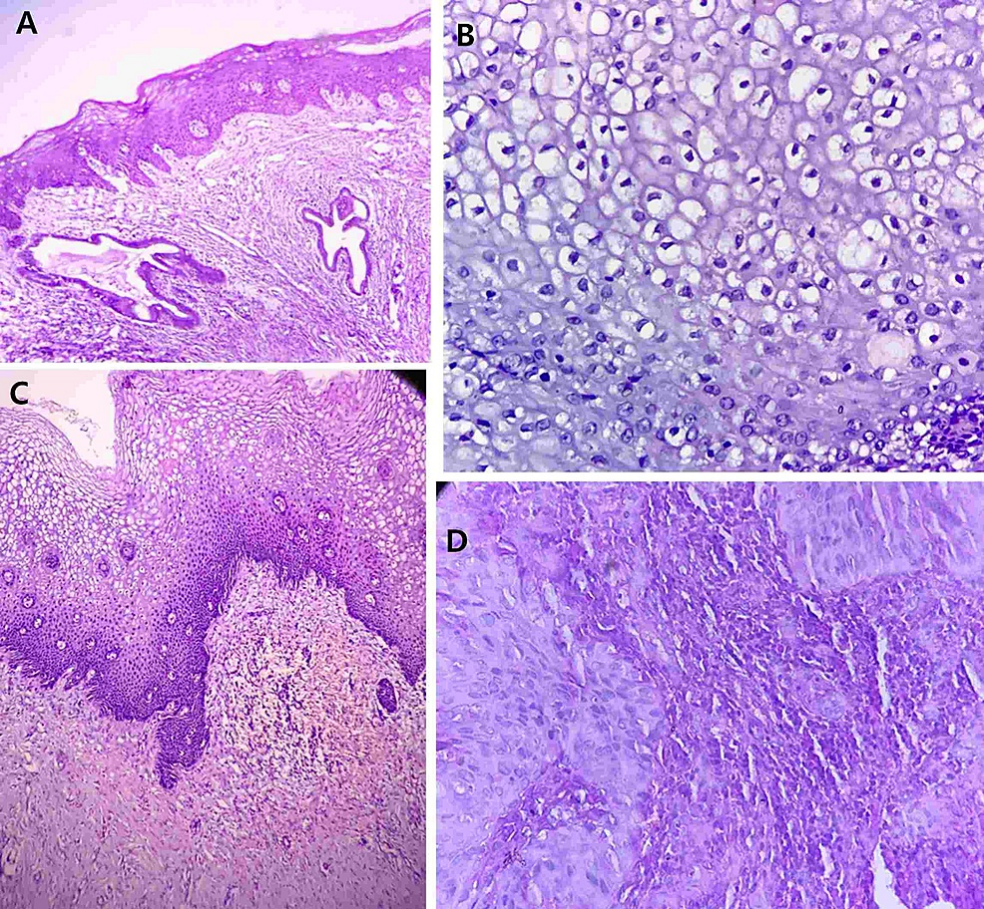
Squamous metaplasia, premalignant, and malignant lesions of the cervix A- H&E stain, 4X: Cervix shows mature squamous metaplasia with underlying endocervical glands. B- H&E stain, 40X: Section shows koilocytosis with enlarged cells, peripheral condensation of cytoplasm, perinuclear halo, irregular nuclear membrane and coarse chromatin. C- H&E stain, 10X: Section from the cervix shows low-grade squamous epithelium with features of loss of polarity involving lower one-third of epithelium, enlarged hyperchromatic nuclei, coarse chromatin and superficial cells with koilocytosis. D- H&E stain, 40X: Section shows nonkeratinizing squamous epithelium with nests of large polygonal cells with hyperchromatic nuclei, and coarse chromatin. These nests are surrounded by a dense lymphomononuclear infiltrate.

A majority (n = 124, 75.6%) presented with excessive menstrual bleeding (one or more of the following: changes of pads more than hourly, the passage of clots, or requirement of blood transfusion). Two-thirds (n = 102, 62%) of the study population had a normal cervix on examination. Twenty-seven women had squamous metaplasia, which was the most common microscopic finding. One-third of para 2 and para 3 women had a diagnosis of chronic cervicitis (Figure 1). Six women (3.6%) had a diagnosis of low-grade squamous intraepithelial lesion (LSIL) and high-grade squamous intraepithelial lesion (HSIL), and one had a malignancy (0.6%) (Figure 2).

Leukorrhea and excessive bleeding were significantly (p < 0.001) associated only with LSIL (Table 2). Chronic cervicitis was significantly associated with a normal cervix (Table 2).

**Table 2:** Comparison of clinical features and microscopic findings with the final diagnoses OR- odds ratio; CI-confidence intervals; LSIL-low-grade squamous intraepithelial lesion; HSIL-high-grade squamous intraepithelial lesion

	LSIL n (%)	p-value	OR (CI)	HSIL n (%)	p-value	OR (CI)	Carcinoma cervix n (%)	p-value	OR (CI)	Polyp n (%)	p-value	OR (CI)	Nabothian cyst (%)	p-value	OR (CI)	Chronic cervicitis n (%)	p-value	OR (CI)
Age (Mean±SD)	50.75±6.80	0.24		53.50±9.19	0.19		47.00±0	0.91		38.07±8.32	0.13		46.40±8.21	0.82		46.07±8.32	0.99	
Young (<40) n=46	0 (0)	0.20	0.966 (0.934-0.999)	0 (0)	0.37	0.983 (0.960-1.007)	0 (0)	0.53	0.992 (0.975-1.008)	2 (1.2)	0.02	1.045 (0.983-1.112)	7 (4.3)	0.99	1.002 (0.449-2.239)	41 (25)	0.89	1.008 (0.896-1.134)
Middle-aged (41-60) n=111	4 (2.4)	0.16	-	2 (1.2)	0.32	-	1 (0.6)	0.48	-	0 (0)	0.03	0.962 (0.912-1.025)	16 (9.8)	0.66	1.178 (0.557-2.490)	99 (60.4)	0.78	1.015 (0.911-1.132)
Elderly (≥61) n=7	0 (0)	0.66	-	0 (0)	0.76	-	0 (0)	0.83	-	0 (0)	0.76	-	2 (1.2)	0.31	0.513 (0.150-1.755)	7 (4.3)	0.350	0.892 (0.844-0.942)
Parity 1	0 (0)	0.69	-	0 (0)	0.78	-	0 (0)	0.84	-	0 (0)	0.78	-	1 (0.6)	0.92	0.911 (0.147-5.663)	5(3)	0.60	1.078 (0.751-1.548)
2	1 (0.6)	0.46	2.234 (0.237-21.035)	1 (0.6)	0.830	0.745 (0.047-11.701)	1 (0.6)	0.24	-	1 (0.6)	0.83	0.745 (0.047-11.701)	10 (6.1)	0.76	1.117 (0.534-2.336)	63 (38.4)	0.89	0.993 (0.894-1.103)
3	2 (1.2)	0.66	0.657 (0.095-4.545)	1 (0.6)	0.76	0.657 (0.042-10.313)	0 (0)	0.41	-	1 (0.6)	0.76	0.657 (0.042-10.313)	9 (5.5)	0.68	1.167 (0.549-2.482)	59 (36)	0.69	0.979 (0.882-1.087)
4	1 (0.6)	0.26	0.302 (0.033-2.726)	0 (0)	0.65	-	0 (0)	0.75	-	0 (0)	0.65		2 (1.2)	0.82	1.158 (0.302-4.438)	12 (7.3)	0.19	1.133 (0.875-1.466)
5	0 (0)	0.69	-	0 (0)	0.78	-	0 (0)	0.84	-	0 (0)	0.78	-	1 (0.6)	0.92	0.911 (0.147-5.663)	6 (3.7)	0.39	0.892 (0.845-0.942)
6	0 (0)	0.82	-	0 (0)	0.87	-	0 (0)	0.91	-	0 (0)	0.87	-	2 (1.2)	0.001	0.142 (0.097-0.207)	2 (1.2)	0.62	(0.849-0.944)
Uterovaginal prolapse (n)	1 (0.6)	0.67	0.618 (0.067-5.723)	1 (0.6)	0.21	0.206 (0.013-3.194)	0 (0)	0.64	-	0 (0)	0.51	-	4 (2.4)	0.87	1.081 (0.402-2.906)	24 (14.6)	0.45	1.055 (0.898-1.239)
Leukorrhea (n)	2 (1.2)	<0.001	0.045 (0.007-0.272)	0 (0)	0.76	-	0 (0)	0.83	-	0 (0)	0.76	-	2 (1.2)	0.31	0.513 (0.150-1.755)	5 (3)	0.10	1.266 (0.790-2.029)
Excessive bleeding (n)	1 (0.6)	0.01	9.300 (0.995-86.917)	1 (0.6)	0.39	3.100 (0.198-48.434)	1 (0.6)	0.56	-	1 (0.6)	0.39	3.100 (0.198-48.434)	19 (11.6)	0.96	0.979 (0.420-2.281)	114 (69.5)	0.08	0.897 (0.771-1.045)
Healthy cervix (n)	1 (0.6)	0.12	4.935 (0.525-46.410)	1 (0.6)	0.72	1.645 (0.105-25.832)	1 (0.6)	0.43	-	1 (0.6)	0.72	1.645 (0.105-25.832)	18 (11)	0.27	0.640 (0.283-1.444)	96 (58.5)	0.01	0.874 (0.771-0.991)
Cervix descended (n)	0 (0)	0.45	-	1 (0.6)	0.10	0.139 (0.009-2.134)	0 (0)	0.70	-	1 (0.6)	0.12	0.139 (0.009-2.134)	3 (1.8)	0.97	1.019 (0.335-3.096)	15 (9.1)	0.02	1.222 (0.944-1.582)
Keratinized cervix (n)	0 (0)	0.46	-	0 (0)	0.60	-	0 (0)	0.71	-	0 (0)	0.60	-	4 (2.4)	0.45	0.688 (0.264-1.790)	17 (10.4)	0.98	1.002 (0.851-1.180)
Rectoceles/cystoceles (n)	1 (0.6)	0.42	0.417 (0.046-3.815)	0 (0)	0.59	-	0 (0)	0.70	-	0 (0)	0.59	-	3 (1.8)	0.97	1.019 (0.335-3.096)	17 (10.4)	0.46	1.062 (0.877-1.287)
Decubitus ulcer (n)	0 (0)	0.62	-	0 (0)	0.73	-	0 (0)	0.80		0 (0)	0.73	-	0 (0)	0.19	-	8 (4.9)	0.94	1.009 (0.796-1.279)
Hyperkeratosis (n)	0 (0)	0.46	-	0 (0)	0.60	-	0 (0)	0.71		0 (0)	0.60	-	4 (2.4)	0.45	0.688 (0.264-1.790)	17 (10.4)	0.98	1.002 (0.851-1.180)
Epidermidization (n)	0 (0)	0.55	-	0 (0)	0.67	-	0 (0)	0.76	-	0 (0)	0.67	-	3 (1.8)	0.41	0.631 (0.218-1.831)	13 (7.9)	0.20	0.887 (0.839-0.939)
Basal cell hyperplasia (n)	0 (0)	0.69	-	0 (0)	0.78	-	0 (0)	0.85	-	0 (0)	0.78	-	1 (0.6)	0.92	0.911 (0.147-5.663)	6 (3.7)	0.39	0.892 (0.845-0.942)
Ulcer (n)	0 (0)	0.62	-	0 (0)	0.73	-	0 (0)	0.80	-	0 (0)	0.73		0 (0)	0.91		8 (4.9)	0.94	1.009 (0.796-1.279)
Endopapillary cervicitis (n)	0 (0)	0.47	-	0 (0)	0.61	-	0 (0)	0.72	-	0 (0)	0.61	-	2 (1.2)	0.60	1.418 (0.364-5.521)	18 (11)	0.13	0.884 (0.833-0.937)
Koilocytosis (n)	1 (0.6)	0.26	0.302 (0.033-2.726)	0 (0)	0.65	-	0 (0)	0.75	-	0 (0)	0.65	-	1 (0.6)	0.33	2.416 (0.351-16.523)	13 (7.9)	0.69	1.038 (0.845-1.275)
Squamous metaplasia (n)	4 (2.4)	<0.001	-	1 (0.6)	0.19	0.197 (0.013-3.055)	1 (0.6)	0.02	-	0 (0)	0.52	-	4 (2.4)	0.941	1.035 (0.386-2.775)	20 (12.2)	0.04	1.251 (0.996-1.572)
Dysplasia (n)	4 (2.4)	<0.001	-	2 (1.2)	<0.001	-	1 (0.6)	<0.001	-	0 (0)	0.78	-	1 (0.6)	0.92	0.911 (0.147-5.663)	0 (0)	<0.001	-

Among the microscopic findings, only squamous metaplasia and dysplasia were significantly associated (p < 0.001) with the final diagnoses such as a low-grade squamous intraepithelial lesion (LSIL), high-grade squamous intraepithelial lesion (HSIL), and chronic cervicitis (Table 2).

## Discussion

During coitus, conception, delivery, and postpartum, the cervix is prone to sexually transmitted infections. Viruses (human papillomavirus, herpes simplex virus 2), and other carcinogens target the cervix and cause invasive cervical carcinoma [1]. Cervicitis is inflammation of the cervix affecting the columnar epithelium of the endocervix and squamous lining epithelium of the ectocervix. Cervicitis may be non-infectious or infectious and can be either acute or chronic based on the duration [6]. Bacteria, fungi, protozoa, and viruses are the infective causes of acute and chronic cervicitis [2]. Acute cervicitis is commonly due to infections such as gonorrhea and chlamydia [6].

Chronic nonspecific cervicitis occurs in women of all age groups between 20 and 69 years but is mostly seen between 41 and 50 years [3, 7]. Most commonly, chronic cervicitis is non-infectious [6]. The major histopathological finding in our study was chronic cervicitis, wherein 89.6% of our patients had this finding. This was similar to the studies performed by Jayakumar (89%) [8], Omoniyi-Esan et al. (82%) [7], Dayal (79.66%) [1]; the studies by Forae and Nwachokor (72.2%) [2], and Purushotham et al. (65.25%) [9] had a much lesser percentage when compared to our study. Sixty percent of chronic cervicitis were seen in middle-aged women, and the mean age of patients with chronic cervicitis was 46.07 ± 8.32 years which was comparable to other study populations. The etiologic (especially, microbiologic) spectrum of chronic cervicitis was not known in our study. The treatment details of such patients with regard to either empirical antibiotics such as doxycycline or azithromycin, or a wait-and-watch policy favored by the treating clinician were not known since we did not have access to outpatient records. Whether such patients received prevention counseling or advice regarding contraception is also not known. Also, follow-up data of resolution of symptoms in such patients were not available. Since most patients with cervicitis may be asymptomatic, diagnosis and treatment are often made late. Screening in sexually active younger individuals is often recommended. Two-fifths of patients in the study by Patel et al. were more than 50 years of age [3]. Omoniyi-Esan et al. had a much older population with chronic cervicitis [7].

Common gynecological symptoms of cervicitis are cervical discharge, pus, and bleeding [6]. The discharge from ectocervix is examined by gram staining, wet mount preparation, and culture to detect sexually transmitted infections due to Candida albicans and Trichomonas vaginalis [6]. At the microscopic level, chronic cervicitis is the most common condition which may lead to pelvic inflammatory disease, endometritis, salpingitis, and chorioamnionitis. Cervicitis can even initiate neoplasia [7]. Histologically chronic cervicitis shows plasma cells, lymphocytes, and histocytes [8]. A collection of lymphocytes forming a germinal center with a thin epithelial lining, seen in the postmenopausal cervix is called follicular cervicitis or chronic lymphocytic cervicitis. Squamous metaplasia is a complication of chronic cervicitis [1]. The most common cause of granulomatous cervicitis is tuberculosis [2]. Chronic cervicitis with koilocytic changes and endopapillary cervicitis were seen in 7.9% and 11% of our patients, respectively. Comparison figures from other studies are 12% (Omoniyi-Esan et al.) and 0.85% (Purushotham et al.), respectively [7, 9]. We did not have facilities for p16 immunohistochemistry staining and human papillomavirus (HPV) DNA polymerase chain reaction, but morphologically they appeared to be due to HPV.

In rural India, uterovaginal prolapse is common due to deliveries conducted at home and prolonged second stage of labor without an episiotomy. In a prolapsed uterus, the ectocervix, due to dryness of the surface epithelium, develop hyperkeratosis and keratinization, which is called epidermidization [1]. Twenty-eight (17.1%) of our patients presented with a mass descending per vaginum, while Dayal et al. had 18.45% patients with a similar complaint [1]. Twenty-four patients (24/28) had findings of chronic cervicitis. On examination, 20 each had a rectocele and descended cervix, respectively. Trauma to the prolapsed cervix leads to erosions and ulcers. Erosions are often accompanied by chronic cervicitis [1]. Nineteen (11.6%) had a keratinized cervix, while nine (5.5%) patients had a decubitus ulcer in our study.

Non-neoplastic tumor-like lesions of the cervix are Nabothian cyst, endometriosis, endocervical polyps, and endocervical hyperplasia [1]. We had 27 such patients with Nabothian cysts (25) and polyps (2). Chronic inflammation of the cervix can lead to the development of a Nabothian cyst [10]. Nabothian cysts are seen in reproductive age groups and do not have clinical significance; grossly, they are small, multiple yellowish to white opaque nodules on the cervix. Microscopically, the cyst is lined by a single layer of flattened or columnar epithelium without atypia or mitosis [10]. Chronic inflammation of the endocervical glands may lead to the development of endocervical polyp [1]. Cervical polyps are seen in multiparous women and arise from the surface of the endocervical canal [1, 11]. It protrudes into the vagina and grossly appears as a red, single, pedunculated, small finger-like growth that arises from the endocervical surface [11]. Histologically, the endocervical glands are dilated in the inflamed, edematous, and fibrotic stroma [1]. The differential diagnoses of cervical polypoidal lesions are leiomyoma, schwannoma, fibroadenoma, adenomyoma, carcinoma, and sarcoma [1, 12]. Fifteen percent in our study had a Nabothian cyst, while it was much rarer in the studies by Forae and Nwachokor (3.3%) [2] and Gupta et al. (7.27%) [13]. Conversely, Forae and Nwachokor (16.3%), Gupta et al. (4.54%), and Kujur et al. (11.65%) had a higher proportion of polyps compared to ours (1.2%) [2, 13, 14].

Viral cervicitis is clinically important [8]. Herpes simplex virus (HSV) and human papillomavirus (HPV) are transmitted sexually [2]. Tissue responses to viral cervicitis are of two types. The response can be either cell degeneration/death or increased mitotic activity leading to neoplasia [7]. Condyloma acuminatum, pre-invasive, and invasive lesions of the cervix are caused by HPV infection. Pre-invasive and invasive lesions of the cervix are LSIL and HSIL and carcinoma cervix, respectively [2]. There were four and two patients with HSIL and LSIL, respectively. The hallmark of HPV infection is cervical epithelium with koilocytic changes [8].

More than 100 types of HPV have been identified [5]. Molecular studies have shown that the most common highly oncogenic HPV types found in invasive carcinoma cervix are HPV-16 and 18. Low-risk type HPV-6 and HPV-11 were seen in genital warts and benign lesions of the cervix [5]. HPV has also been detected in healthy women with benign cervical cytology [5]. In India, women in the age group of 26-35 years generally develop HPV infection; in developed countries, it develops a decade later [5]. According to current concepts of cervical carcinogenesis, there are three steps of HPV infection: preinvasive lesions, high-grade, and invasive lesions. More than 95% of LSIL or low-grade dysplasia spontaneously resolve and become HPV DNA negative [5].

Patel et al. had shown that the most common lesion among women of age groups 31-40 years was cervical intraepithelial neoplasia (CIN) [3]. Based on the architectural disturbances and cellular atypia, CIN is classified into three grades [6]. The histological features of CIN I are: epithelium of lower third or less, shows no stratification or cytoplasmic differentiation, lack of defined borders of cells, nuclear crowding, and an increased nucleocytoplasmic ratio [15]. Sometimes, aberrant keratinization and mitotic figures are seen. The nuclei of the epithelium are pleomorphic, enlarged, hyperchromatic with prominent nucleoli, and have filamentous/granular chromatin [15]. The features of CIN II are similar to CIN I except for the presence of non-stratified undifferentiated cells with increased nucleocytoplasmic ratio, and mitotic figures are present beyond the lower one-third and spare the upper third of the epithelium. CIN III shows non-stratified, undifferentiated, basaloid cells, with the nucleus showing pleomorphism, crowding along with mitotic figures in the upper third of epithelium [15]. This classification has poor reproducibility and hence the Lower Anogenital Squamous Terminology (LAST) project reclassified CIN I as LSIL. CIN III is reclassified as HSIL. CIN II that are p16 stain positive are referred to HSIL; otherwise, they are referred to LSIL [16]. We did not have p16 staining in our institution, but morphologically the lesions corresponded to HSIL. We had only two patients with HSIL in our study.

High-risk factors for the development of squamous cell carcinoma include age, coitus, poor genital hygiene, low socioeconomic status, multiple sex partners, use of oral contraceptives, smoking cigarettes, infection with HSV type-2, HPV, immunosuppression, and abnormal pap smear [17]. Cervical cancer presents with bleeding after and before coitus, back pain, abdominal pain, leukorrhea discharge, malodor in the vagina, urinary urgency, and renal failure [4, 18]. The commonest site for carcinoma is the squamo-columnar junction [4, 17]. According to the grading system of Broder, squamous cell carcinoma is classified into well-differentiated, moderately differentiated, and poorly differentiated carcinoma [19]. There was only one patient with carcinoma cervix in our study that was well differentiated. About 90% of carcinomas are squamous carcinomas, and the remainder is adenocarcinoma [15].

Visual inspection, cytology, and HPV testing are the three screening modalities of HPV infection [5]. In comparison between visual acetic acid (VIA), and conventional Pap test, the cervical smear is considered better, while biopsy remains the gold standard. For a better cytohistological correlation of cancerous and precancerous lesions of the cervix, a colposcopic-directed biopsy is used [17].

Limitations

This was a retrospective study, and clinical and socioeconomic details of some patients were unavailable in the case files. Similarly, follow-up data of patients with premalignant lesions could not be accessed to analyze the progression of the lesions. We did not have details as to whether the deliveries had been conducted at home, by midwives or other trained personnel. Koilocytosis in our samples were not investigated further for HPV infection since this facility was not available in our institution.

## Conclusions

Most studies involving rural populations have involved the knowledge, attitude, and practices of the study cohort. An overwhelming majority of our study population had pathological findings of chronic cervicitis, the etiology of which is fully not known. Since many patients are often asymptomatic, diagnosis and treatment are delayed. Low socioeconomic status, poor hygiene, poor obstetric care, and ignorance in our rural cohort may have been responsible for the increased incidence in this cohort. Cervicitis (mainly due to viruses like HPV and HSV-II) may even initiate neoplasia, and it is important that such patients be screened for and followed up for clinical, microbiological, and pathological findings. Since excessive bleeding and leukorrhea are associated with HSIL, patients with these complaints should be fully investigated to prevent invasive cervical carcinoma.
